# Meralgia Paresthetica: A Case Report With an Update on Anatomy, Pathology, and Therapy

**DOI:** 10.7759/cureus.13937

**Published:** 2021-03-17

**Authors:** Hassan Kesserwani

**Affiliations:** 1 Neurology, Flowers Medical Group, Dothan, USA

**Keywords:** lumbar plexus, peripheral nerve disorders

## Abstract

Meralgia paresthetica, a condition characterized by tingling, numbness, and burning pain in the lateral aspect of the thigh, is caused by compression of the lateral femoral cutaneous nerve. The incidence of meralgia paresthetica increases with obesity and diabetes. The unique anatomy of the nerve that tunnels through the inguinal ligament predisposes it to inflammation, trauma, and entrapment. The pathology of meralgia paresthetica parallels that of entrapment neuropathies but with additional inflammatory overlay in certain instances. The clinical diagnosis is relatively simple due to its unique clinical features. The prognosis is generally excellent, and the treatment is straightforward that includes peripheral nerve blocks, neurectomy, nerve decompression, and pulsed radiofrequency neuromodulation. This current case of meralgia paresthetica highlights the salient clinical symptoms and signs. We have also described the electrophysiological studies of the lateral femoral cutaneous nerve, its anatomical variations, and the associations of meralgia paresthetica with bariatric surgery, critical care patients, tight clothing, pregnancy, and posterior spine surgery. We have also outlined the current treatment strategies.

## Introduction

The lateral femoral cutaneous nerve of the thigh (LFCNT) arises from the lumbar L2 and L3 nerve roots, merging with the lumbar plexus and piercing and emerging from the psoas muscle. It usually threads through a tunnel in the inguinal ligament, anywhere from 1 to 7 cm anteroinferior to the anterior superior iliac spine (ASIS). The LFCNT usually bifurcates below the inguinal ligament into an anterior and posterior division. It is a purely sensory nerve supplying sensation to the anterolateral surface of the thigh. It is prone to trauma, entrapment, and inflammation at the inguinal ligament level, where disease here is referred to as meralgia paresthetica; by definition, a mononeuropathy. This leads to numbness, tingling, burning, prickling sensations, and dysesthesias to touch or rub over the anterior and/or lateral thigh [1].

Nerve preparations of the LFCNT following neurectomy reveal multifocal, large-diameter myelinated nerve fiber loss, regenerative clusters, relative preservation of small fibers, and perineural thickening reflecting fascicular compression of the nerve. Occasionally, perivascular (intraneural and epineural) inflammation reflects an inflammatory process [2].

Meralgia paresthetica is independently associated with obesity, advancing age, and diabetes. Its incidence is 32.6 per 100,000 patient-years. The incidence increases seven-fold in patients with diabetes, and meralgia paresthetica patients are twice as likely to develop diabetes, a risk factor independent of obesity [3]. In the Rotterdam study, in a primary care setting for nine years, the incidence rate of meralgia paresthetica was 4.3 per 10,000 patient-years. The odds ratio (OR) of developing meralgia paresthetica with pregnancy was 12 (95% confidence interval [CI] 1.2-118.0). This compares with an OR of 7.7 for carpal tunnel syndrome (95% CI 1.9-31.1) [4].

Electrophysiologically, in a study of 131 cases, measurement of the sensory nerve action potential (SNAP) of the LFCNT (abnormal SNAP: <3 µV) is less sensitive (73.3%) than a side-to-side SNAP amplitude ratio (abnormal ratio: >2.3), with a sensitivity of 98.3% [5]. The diagnosis of meralgia paresthetica is relatively simple, as it is a purely sensory disorder, and the LFCNT has a relatively well-circumscribed dermatomal distribution whereby sensory symptoms never extend below the knee. Meralgia paresthetica is usually easily distinguished from lumbar radiculopathy and lumbar plexopathy by history and examination. Useful clinical signs include tenderness medial to the ASIS, dysesthesia to rub along the anterolateral thigh, and aggravation of sensory symptoms by leg extension. There is also an absence of weakness and low back pain. Definitive treatment includes peripheral nerve blocks of the LFCNT, neurectomy, nerve decompression, and pulsed radiofrequency stimulation [6].

## Case presentation

A 44-year-old-man presented with a one-month history of burning, tingling, aching, and shooting pain of the anterolateral aspect of the right thigh. He denies any back pain, weakness, or radicular symptoms. The discomfort increases at night when lying in bed and with prolonged standing and walking. Bed sheets rubbing on his right thigh is bothersome. He has gained 15 pounds over two years.

His past medical history is significant for hypertension, for which he takes losartan 100 mg daily. His height is 6 ft, 1 inch, and he weighs 310 pounds with a body mass index of 40.9 kg/m^2^. Gait with heel-walking and toe-walking was normal. He can stand up from the seated position with his arms folded. Power testing of the lower extremities, including hip flexion, thigh adduction and abduction, knee extension and flexion, ankle dorsiflexion, and plantar flexion with toe flexion and extension, graded 5/5 by the medical research council scale and symmetric. Deep tendon reflexes were lively at the knees and ankles and symmetric in the legs.

There was tenderness to deep percussion, approximately two finger-breadth medial to the right ASIS. Tingling of the right thigh was elicited by right leg extension at the hip. Sensory examination revealed dysesthesia to gentle rubbing along the anterolateral surface of the right thigh. Vibration, touch-pressure, and joint-position sense were preserved at the big toes bilaterally.

**Figure 1:**
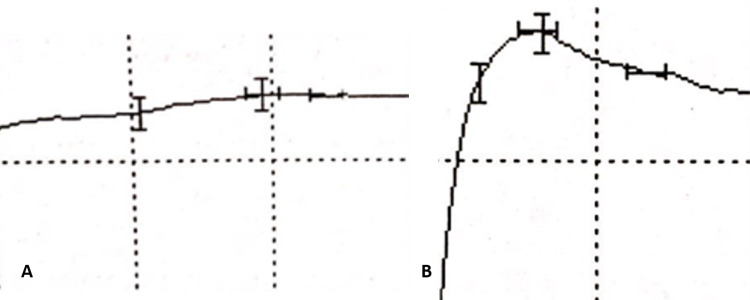
Nerve conduction study. (A) Absent right lateral femoral cutaneous nerve SNAP of the thigh. (B) Normal left lateral femoral cutaneous nerve SNAP of the thigh. Abscissa: 5 msec, Ordinate 20 µV msec, milliseconds; SNAP, sensory nerve action potential

A nerve conduction study revealed normal bilateral peroneal and tibial motor amplitudes and velocities, with borderline amplitudes of the peroneal and SNAP amplitudes bilaterally. The left LFCNT SNAP was normal at 5.2 µV (reference standard, >3 µV), and the right LFCNT SNAP was absent, confirming the diagnosis of a right meralgia paresthetica (Figure 1).

A high-volume peripheral nerve block of the right LFCNT with 4 mL xylocaine 1%/8 mg dexamethasone was performed with immediate relief. The injection was placed subcutaneously, 3 cm anteroinferior to the ASIS at the site of maximum tenderness. At his one-month follow-up, the patient was symptom-free. The patient was advised to lose weight by referral to a nutritionist and follow-up with his family doctor to be screened for diabetes. He was also counseled about other potential therapies, such as a neurectomy or nerve decompression, should symptoms return.

## Discussion

The LFCNT is a branch of the lumbar plexus emanating from the L2 and L3 nerve roots. Its mean distance as it enters the inguinal ligament is 1.9 cm medial to the ASIS. This mean distance is 0.99 cm in South Americans and 2.31 cm in European and North American populations. A safe distance for an incision for inguinal hernia surgery is at least 3 cm medial to the ASIS. The nerve usually enters the thigh overlying the surface of the sartorius muscle, where it bifurcates into its anterior and posterior divisions [7]. The three main patterns of division of the LFCNT are presented in Table 1 [7].

**Table 1:** Variation of LFCNT as it enters the thigh LFCNT, lateral femoral cutaneous nerve of the thigh; ASIS, anterior superior iliac spine

Variation of LFCNT	Percentage [7]
Single branch exits pelvis anterior to ASIS, under or through the inguinal ligament and medial to the sartorius muscle. Bifurcation below inguinal ligament	86.8
Bifurcation within pelvis	11.8
Trifurcation within pelvis	1.2

In a study of 29 cadavers, the LFCNT was located up to 7.3 cm medial to the ASIS and up to 11.3 cm distally on the sartorius muscle before bifurcating, which is a wide variability [1]. In a study of 52 anatomical dissections, five modes of variation of exit of the LFCNT from the pelvis were described. When the LFCNT approaches the inguinal ligament, it makes a 90° sharp turn. Furthermore, the nerve is anchored at the exit from the psoas muscle and its entry into the inguinal ligament and occasionally anchored at its entrance into the sartorius tendon. Rarely, it passes posterior to the ASIS. These patterns predispose the nerve to trauma. These variations are summarized in Table 2 [8].

**Table 2:** Patterns of exit of LFCNT from the pelvis in relation to the ASIS LFCNT, lateral femoral cutaneous nerve of the thigh; ASIS, anterior superior iliac spine

Pattern of exit from pelvis	Percentage [8]
Posterior to ASIS	4
Anterior to ASIS through inguinal ligament tunnel	27
Medial to ASIS through sartorius tendon	23
Deep to inguinal ligament (between sartorius and iliopsoas)	26
Most medial overlying iliopsoas and anastomosing with genitofemoral nerve	20

Besides using empirical therapy with non-steroidal anti-inflammatory drugs and gabanoids such as gabapentin, the mainstay of therapy is local peripheral nerve blocks with local anesthetics and/or steroids, nerve decompression, neurectomy, and pulse radiofrequency ablation [9]. In a meta-analysis of four studies, 30 of 157 patients showed improvement or full resolution (83%) with high-volume peripheral nerve blocks of the LFCNT. Surgical therapy is recommended after failure of peripheral nerve blocks. Surgical therapy with a neurectomy demonstrated efficacy in 45 of 48 cases (94%: three studies), and 264 of 300 cases demonstrated efficacy with LFCNT decompression (88%: nine studies), approximately equal efficacy between both techniques [6,10].

Pulsed radiofrequency (PRF) neuromodulation applies electromagnetic energy in the radiofrequency range in a nerve's vicinity under ultrasound guidance. It alters nerve function by reducing inflammatory mediators, such as tumor necrosis factor-alpha and interleukin-6 and neuromodulation (suppress C-fiber activity and subsequent dorsal horn inhibition). In contradistinction, continuous radiofrequency ablation applies enough energy (heat) to destroy the nerve (thermocoagulation and necrosis). The comparison between PRF neuromodulation and continuous radiofrequency radioablation is summarized in Table 3 [11,12].

**Table 3:** Comparison between pulse radiofrequency neuromodulation and continuous radiofrequency ablation

	Continuous radiofrequency ablation	Pulsed radiofrequency neuromodulation
Temperature (°C)	90	42
Duration	90 seconds	Pulsatile; 20 millisecond pulses every 0.5 seconds
Mechanism	Thermocoagulation and tissue necrosis (ablation); electric field alters C-fiber activity and dorsal horn inhibition (neuromodulation)	Neuromodulation more than tissue injury (ablation)
Advantages		Longer duration of pain relief / less painful procedure/ lower risk of deafferentation pain
Disadvantages	More painful procedure/risk of deafferentation pain	

In a study of 11 patients with meralgia paresthetica treated with PRF, two-thirds of patients achieved complete pain relief, and one-third of patients achieved satisfactory relief with remission at their six-month follow-up [13]. Besides diabetes and obesity, there are tight associations between certain other medical conditions and meralgia paresthetica (Table 4) [14-19].

**Table 4:** Disease associations with meralgia paresthetica COVID-19, novel coronavirus disease-2019; ICU, intensive care unit; BMI, body mass index; ND, not disclosed

Study	Patient cohort	Study population (n)	Proportion with meralgia paresthetica	Characteristics
Christie et al. [14]	COVID-19 ICU patients	51	33%	Bilateral meralgia paresthetica: prone positioning in 47%, diabetes in 23%, BMI >30 kg/m^2^ in 37%
Macgregor and Thoburn [15]	Bariatric surgery postoperative patients	ND	11 patients	Extrinsic pressure from abdominal retractor accounted for some cases, not all
Yang et al. [16]	Posterior spine surgery	252	23.8%	Higher BMI, longer surgical time, and higher percent of degenerative spinal disorder
Ziaie [17]	Transfemoral coronary angiography	1550	0.00322%	
Edelson and Stevens [18]	Pediatric population	ND	20 patients	50% were bilateral, most lesions required surgical decompression
Moucharafieh et al. [19]	Fashion with tight low-cut trousers	ND	12 patients	

## Conclusions

Meralgia paresthetica is a relatively common condition with easily recognizable features that usually allows the clinician to make an immediate or "spot" diagnosis. Its treatment is also simple, with an excellent prognosis. The anatomy of the LFCNT is well defined, and its variability is well documented. The various clinical scenarios of its occurrence in patients with diabetes, obesity, pregnancy, those in intensive care unit settings, and posterior spine surgery and bariatric surgery recipients have been increasingly recognized. Pathologically, the findings parallel what one finds with peripheral nerve entrapment with an inflammatory overlay in some instances, as seen with diabetes. Increasing recognition of meralgia paresthetica will obviate the need for expensive and unnecessary testing.
